# Gender inequalities in the sale of handmade corn tortillas in central Mexican markets: before and during the COVID-19 pandemic

**DOI:** 10.1186/s42779-022-00119-6

**Published:** 2022-02-19

**Authors:** Alma Lili Cárdenas-Marcelo, Angélica Espinoza-Ortega, Ivonne Vizcarra-Bordi

**Affiliations:** Instituto de Ciencias Agropecuarias y Rurales, Universidad Autónoma del Estado de México, Instituto Literario No. 100 oriente, Colonia Centro, Toluca, Mexico

**Keywords:** Gender, Native maize, Markets/*tianguis*, Inequality, Mazahua people, Central Mexico, COVID-19

## Abstract

**Objective:**

The objective of this study was to analyze gender inequalities and intersectionality experienced by rural-indigenous women who produce and sell native maize tortillas at three different markets-tianguis in central Mexico, facing the COVID-19 pandemic.

**Methods:**

This was a qualitative study based on 36 in-depth interviews before pandemic (2018), as well as 16 interviews during pandemic (2020) of women engaged in this work.

**Results:**

Making corn tortillas by hand is one of the culturally assigned gender roles in the indigenous population of the Mazahua region, which is why their sale in local markets as a female strategy to have access to income for household sustenance has been widely by the communities. The configuration of the different market-space for the sale of handmade tortillas, reflects the inequalities of gender and intersectionality (ethnicity, class, age, family life cycle and education levels). The women in conditions of poverty, landlessness, and with school-age children, have met greater disadvantages in continuing to sell tortillas in the face of the experience of pandemic restrictions.

**Conclusions:**

The women who were already disadvantaged by their intersectional relationships continue to experience the same inequalities that conditioned their position in the marketplaces before the pandemic, sustaining a marginal but constant market.

## Introduction

It has been recognized that rural women worldwide are not only responsible for feeding their families and contributing to the production of food, but also for helping preserve much of the biodiversity of their localities. However, few are represented at the managerial level and in decision-making in the public spaces in which they participate [1], such as marketplaces where they carry out part of their economic and commercial activities that enable them to support their families [2]. The consumption of ethnic foods is a rapidly growing trend both in contexts of food globalization [3] and in gastronomic movements. Still, due to the cultural patrimonialization of national cuisines [4], the role of rural women who preserve these foods is not often valued outside global discourses [5]. Furthermore, the 2020 global health emergency caused by COVID-19 plunged all societies into a new crisis scenario, so that a number of questions about life and human dignity are being asked on different academic, social and political forums [6]. One of them is the role of women in the preparation of basic foods, many of them of ethnic origin. Although this occupation is undoubtedly considered an essential activity in families and local markets, when faced by health crisis scenarios, it may or may not put rural, poor and indigenous women at a disadvantage, or deepen the inequalities that have historically subordinated them to extensive systems of dominance [7].

**Fig. 1:**
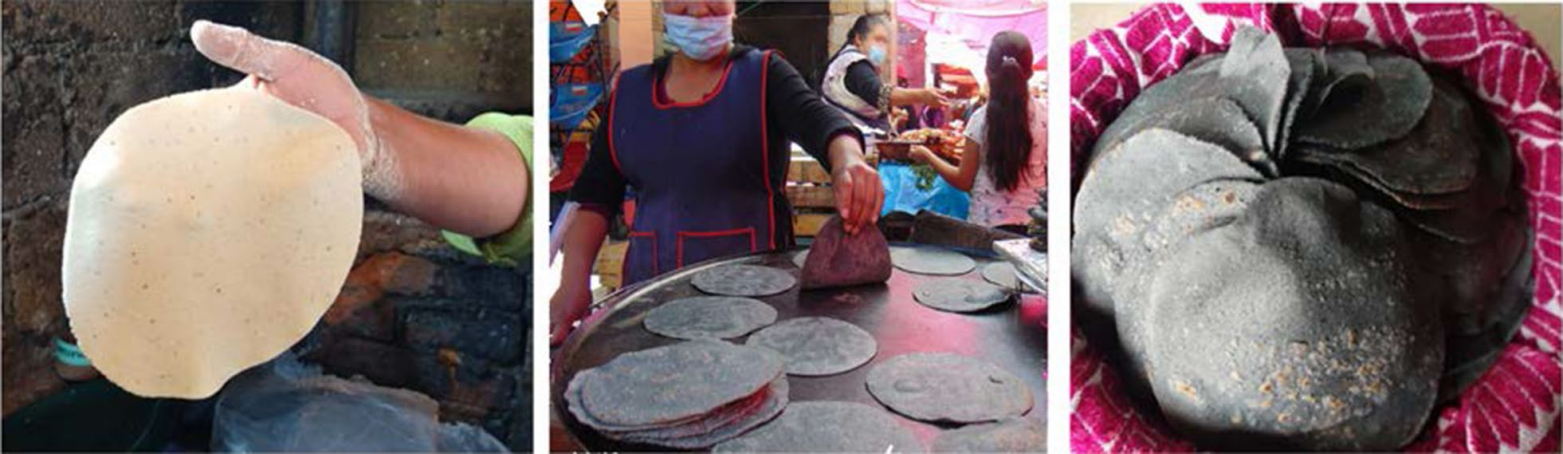
A tortilla is a circular flatbread (12–18 cm in diameter and 1–2 mm thick). It is made of nixtamalized *masa*, cooked on a (clay or metal) *comal*, where it is turned three times until puffed and ready for consumption. It is served as an accompaniment to food, or consumed as part of other simple or more elaborate dishes [9]. Photographs by Alma Lili Cárdenas Marcelo (2020)

In Mexico, maize is a food of ancestral ethnic origin. Besides being the basic cereal in the Food Security and Food Sovereignty policy, it is the most deeply rooted grain in the Mexican food culture due to its diversity, as well as its culinary, ecological, spiritual, and nutritional versatility [8]. With the recognition of traditional Mexican cuisine as Intangible Cultural Heritage of Humanity [9], maize has taken on new prominence in which handmade tortillas have played a pivotal role (Fig. 1). Different maize varieties are used by rural women for an artisanal production based on popular knowledge and assigned gender roles [10].

**Fig. 2:**
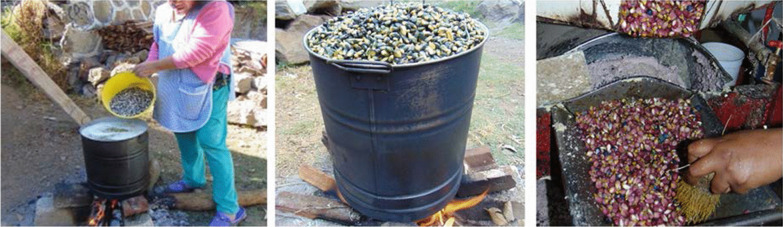
Nixtamalization is the technological process for the preparation of *nixtamal* (from Nahuatl *nixtli*, ash/lime, and *tamalli*, *masa* or corn dough). First, dry maize kernels are cooked in a water-lime solution near the boiling point, until the kernels’ hulls can be easily removed. The new mixture is left to rest for 6–18 h, so that the maize kernels are hydrated with the alkaline solution. Subsequently, the nixtamalized kernels are wet milled in order to obtain fresh *masa* [12]. Photographs by Alma Lili Cárdenas Marcelo (2020)

Throughout history, tortillas have been the main form of maize consumption in Mexico. Tortillas are high in carbohydrates; they provide energy, as well as dietary fiber, proteins, and some vitamins. Through the nixtamalization process (Fig. 2), tortillas are also a source of calcium, potassium, phosphorus, and niacin, which improves the digestibility of proteins [11].

Tortillas emerged in the Late Formative stage (400 BC to 100 AD) [13]. Since Precolumbian times, tortilla making was carried out at home by Mesoamerican women, regardless of social class. It thus constitutes a female legacy, and this activity often characterized the idea of what it was to be a “good woman” [14]. Another way of obtaining tortillas was the *tianguis* (from Nahuatl *tianquiztli*, meaning “market”), where women offered them in woven baskets in order to keep them warm [15].

Until 5 decades ago, native maize varieties were used in the production of tortillas, which were exclusively handmade by rural and indigenous women or else prepared in *tortillerias* (tortilla bakeries), using basic technology. Starting with the Mexican urbanization and industrialization process in the 1960s, tortillas have also been produced in different food systems, gaining space in national and international markets. Despite this, the handmade tortilla market continues to coexist, albeit with great disadvantages with regard to the multinational maize and tortilla agroindustry that favors intensive monoculture of agrochemical-dependent hybrid maize [16].

**Fig. 3:**
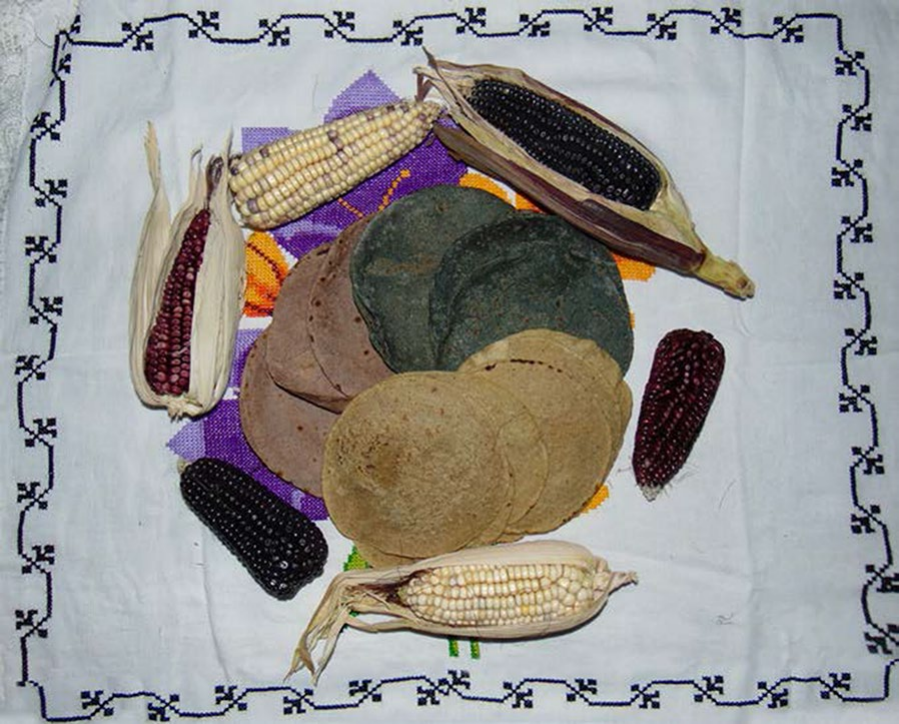
Variety of tortillas made from a native maize diversity. Photograph by Alma Lili Cárdenas Marcelo (2020)

The weak but growing demand for handmade tortillas by the urban middle and upper classes is due to the emergence of consumers who are conscious of the importance of rescuing native maize varieties, which are associated with healthy ethnic foods and reinforce their Mexican identity [17] (Fig. 3). Despite this movement, the work of the women who prepare and sell these tortillas is undervalued by keeping the selling price low. Furthermore, they are relegated to small spaces in marketplaces and *tianguis* [18]. Even so, they have been a source of economic development for the rural population [19], due to the fact that their main input is native maize produced by small farmers [20].

As a staple food, maize is still produced in different systems. Consequently, tortillas continue to be present in the diet of Mexican families, before and during the pandemic. The uncertainty of selling handmade tortillas at marketplaces and *tianguis* has surely generated new questions about the strategies that rural women carry out to supply these markets, formerly frequented by groups of regular consumers [21].

Corn tortillas are consumed to accompany the meals of the Mexican population and are used to make tacos and more than 300 dishes throughout the several cultural regions of the country. Others foods derived from nixtamalized corn dough are also considered ethnic foods and are sold like tortillas in local markets, *tianguis* and on the streets (e.g., *tamales, atole, gorditas, tlacoyos, tostadas, tlayudas,* cookies, etc.) [10].

Recently, the different types of handmade tortilla markets have begun to be studied [19], as well as the gender relations that develop in these spaces [5, 18, 21], where gender inequalities become visible. The unfair competition imposed by the growth of new establishments selling semi-industrialized tortillas made from hybrid corn flours. The repercussions of higher sales volumes of these types of tortillas are: (a) displacement of rural women from their local markets and (b) new social spatial segregation [5, 20, 21]. Nonetheless, little has been done to address their intersectional links with other categories of subordination: class, ethnicity, age, education levels and, family life cycle. In the face of the health crisis caused by the global pandemic, the question arises as to how these inequalities and their intersectionality are deepened or not in this crisis. The purpose of the study is to answer this question, to know how these women came to confront the restrictions caused by the COVID-19 pandemic. Diverse marketplaces and *tianguis* in a medium-sized city renowned for its regional and national trade at Mazahua Region in Central Mexico, are considered as a case study. The Mazahua region is one of the five indigenous regions situated in central Mexico (Otomi-Querétaro e Hidalgo, Nahuatl, Otomí-Estado de Mexico y Chocho-Mixteca-Popoloca) [13, 22].

### Gender inequality and intersectionality an approach

Gender inequalities are understood from different approaches. The UN refers to the disadvantages suffered by women compared to men in the social, cultural, political, knowledge, spatial, economic and environmental spheres [23]. For some Latin American approaches, these disadvantages are consequences of the structures of colonial-patriarchal domination imposed on poor countries or less-developed countries. They propose to study the roots that give rise to inequalities [23] in order to understand how these multidimensional disadvantages are produced in the lives of indigenous and rural women.

Among these roots, they point out those derived from the forms of racial domination to which indigenous peoples were subjected; the division of social classes that came with colonialism and the progress of capitalism; and the cultural assignments of traditional gender roles. These forms of domination caused indigenous women to be doubly excluded from political and economic access to a free and dignified life [23]*.*

A few decades ago, feminist studies had reported the triple oppression of sexism, classism, and racism experienced by women [24]. However, the existing theoretical framework lacked the analysis of the interaction of gender with other ontological dimensions that analyze social identity structures through interdependent mechanisms of subjection and resistance [25]. Intersectional analysis opened the opportunity to include all the voices oppressed by diverse subordinate categories (gender, class, race, ethnicity, age, religion, sexuality) [23] to denounce the several social inequalities (exclusion and discrimination) to which they have been subjected [23].

Women who sell ethnic foods under these conditions have begun to be studied in anthropological and sociological research [2, 26, 27]. To contribute to the knowledge of differentiated strategies that rural women follow to insert themselves in local markets, gender intersectionality (class, ethnicity, age) with other social categories that are interlinked with gender relations (family life cycle and, levels of education) must be considered.

The Class category is defined by their peasant lifestyles with or without access to land to produce maize for self-consumption. Generally, their production only supplies up to 6 months of their food needs [20]. This condition forces them to look for other sources of income to buy food [5]. This category necessarily implies the peasantry, where the processes in the struggle for access to land and the defense of native seeds become essential elements to understand social inequalities [20, 21].

The ethnicity category is defined in the relations of domination imposed in colonization and non-indigenous modernity-civilizing progress, through mechanisms of social exclusion and discrimination and intolerance to the identities of native peoples [23].

Age is understood as belonging to a generational group. The family life cycle is positioned as an important analysis category to understand the workloads of women in the home, the period of biological reproduction and childcare. Depending on it, women manage their time to do income-generating jobs [28].

The level of education is considered more as a social condition that generates inequality than an analytical category [29]. However, from an intersectionality view [30], people who can access better levels of education and cultural capital have greater capacities to confront civilizing crises, such as the health crisis exposed in the pandemic [31, 32].

An analysis from this perspective also allows recognizing women’s work as the basis for the continuity of food culture [10]. It highlights these women’s key role in looking for food security in rural and community areas, showcasing the vulnerability of indigenous women who prepare and sell ethnic products in the face of global crises such as the COVID-19 pandemic.

## Methods

A qualitative study [28] was performed in Ixtlahuaca, State of Mexico, a municipality of ethnic Mazahua origin in Central Mexico, located at an altitude of 1800–3000 masl (meters above sea level). The climate is humid temperate, with intermountain valleys that favor the traditional systems of native maize cultivation with its diverse races and varieties [33]. Ixtlahuaca is a municipality of great interconnectivity in the Mazahua region which allows for wide trade dynamics among locations and cities. There are three markets that converge in space and time. One of them is recognized as the largest in the region: The Traveling *Tianguis* (weekly street markets on strategic locations in town’s streets). It goes back to more than 100 years. The diversity of ethnic food is great, and it includes mushrooms, insects, reptiles and Pigweed [13, 22].

The results of a first qualitative study done from October 2016 to July 2018, carried out with the objective of getting to know the feminine strategies in the making and selling of artisan corn tortillas in these three unique markets in Ixtlahuaca [34], led to the concern of finding out if these strategies had had to adapt facing the COVID-19 pandemic crisis. Therefore, we asked ourselves: which were the new problems that those women faced to continue the making and selling of artisan tortillas?

In that first study, all the women that sold tortillas in those three markets were initially identified and invited (73 women). Thirty-six of them accepted to participate in the investigation. Accompanied by ethnographic research technics (participating observation, field work notebook and photographic file), each one of them accepted to talk in Spanish. An in-depth interview was carried out. In average it lasted between 10 and 15 h (an interview was completed in 5 to 8 visits). The audio was recorded and transcribed manually. The interviews dealt with the following subjects, besides of the sociodemographic identification file (Table 1): (a) motivations to occupy oneself in the making and selling of artisan tortillas in the markets; (b) problems, insertion strategies and permanence in the markets; (c) perception, practices and significance of the native maize throughout their lifetime; (d) traditional wisdom and generational knowledge transmissibility; (e) processes of elaboration and commercialization of tortillas (Table 2); distribution of domestic and productive work, and; (f) social limitations to the access of the means of production (land, seeds, levels of education and freedom of mobility).

**Table 1:** Social characteristics of women who produce and sell handmade tortillas at the marketplaces of Ixtlahuaca *Source*: fieldwork 2018–2019

Characteristic	*La Placita*		Travelling *Tianguis*		Municipality market	
Total women interviewed in 2018	16		12		8	
Ethnic affiliation	Speaker of Jñatjo-Mazahua language		Passive user of Jñatjo-Mazahua language (understands it, but does not speak it)		Of Jñatjo-Mazahua origin, but neither speaks nor understands the language	
Age range	33–73		30–57		23–51	
Average age (years)	57.5		41		38.3	
Marital status	In a relationship	14	In a relationship	7	In a relationship	4
	Widowed	2	Single/divorced parent	4	Single/divorced parent	3
			Widowed	1	Widowed	1
Levels of education^a^	Without education or incomplete primary school	11	Primary school	8	Primary school	2
	Primary school	2	Lower secondary school	3	Lower secondary school	5
	Lower secondary school	3	High school	1	High school	1
Family life cycle	Empty nest (no dependent children at home)		Consolidation, with children between 3 and 17 years old. No childcare support		Consolidation, with children between 3 and 17 years old, with childcare support	

^a^In Mexico, on average, 6 years are studied in primary school, 3 years in secondary and, 3 years in high school

**Table 2:** Tortilla production at the different marketplaces before and during the COVID-19 pandemic

Marketplace			
Process	*La Placita*	Travelling *Tianguis*	Municipality market
*Before pandemic period*			
Type of maize used	Mainly native	Native and hybrids	Only hybrid
Access to maize	Mostly from own cultivation	Mostly locally bought	Bought at wholesale stores
Place of preparation	At home	On the street	At the store
Sale location	Sidewalk and street corners	On the street	At the store
Production process	Nixtamal, local semi-industrial milling, tortilla press, steel *comal* and wood stove (photo 1)	Nixtamal, local semi-industrial milling, tortilla press, round steel *comal* and gas stove (photo 2)	Nixtamal, local semi-industrial milling, tortilla press, rectangular steel *comal* integrated into a gas stove (photo 3)
Types of tortillas	White, blue, occasionally pink (with or without whole wheat flour)	White, blue, occasionally pink and yellow (with or without whole wheat flour)	White, blue (with or without corn flour)
Characteristics of the tortilla	Big and thick (18 cm diameter, 40 g)	Regular artisanal (16 cm diameter, 35 g)	Regular standard (15 cm diameter, 33 g)
Sales format	Dozen	Dozen or weight	Mainly weight
Sales price by format in August 2018	Warm and retail purchase: 0.66 USD Cold and wholesale purchase: 0.50 USD	Dozen, freshly made, and retail purchase: 0.66 0.85 USD per kg	Freshly made, retail purchase: 0.70 to 0.80 USD per kg
Sales volume	35 kg per week	45–50 kg per day	150–200 kg per week
Type of consumers	Local and intermediary consumers	Local and regional consumers	Local consumers
Jobs created	85% of sellers employ one female helper The remaining ones employ 2 or 3 people	60% of sellers employ two women 40% employs 3 or 4 people	40% of sellers employ 2 people 60% employ 3 people
*Beginning pandemic*			
Sale price by format in the year 2020	0.86 USD a dozen. Warm, retail price	0.72 USD a dozen. Retail freshly made 0.86 to 0.96 USD per kg	0.86 to 0.96 USD per kg. Retail, freshly made
Sales volume	50 kg per week	–	100 kg per week
Type of consumers	Local consumers		Local consumers
Pandemic COVID-19 situation	Enabled without restrictions: increase in sales and 50% reduction in the number of women selling them	Disabled	Enabled during the red code, but with reduction in sales. Loss of employment and increase risk of business closure due to elevated cost of renting premises y administrative expenses

The second stage of the investigation took place from June to August 2020. Our search was for the 36 women who had participated in the previous study, but only 16 of them were found, and they were interviewed again. It is worth mentioning that no new saleswomen were recognized during this field work. The direct interviews were structured using open questions related to the difficulties encountered in making a living by doing the same activity within the context of government-imposed confinements and restrictions. Adding both interviews, the narratives were coded by intersectional categories to differentiate coping strategies of women that continue to work in the markets.

### Marketplaces of Ixtlahuaca

**Fig. 4:**
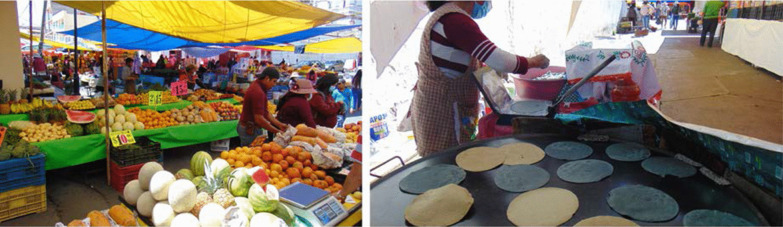
The weekly wandering *tianguis* has taken place on Mondays since the start of the twentieth century [33]. It is the biggest open-air market in the indigenous Mazahua (Jñatjo) ethnoregion. Woman selling tortillas in this *tianguis.* Photographs by Alma Lili Cárdenas Marcelo (2020)

**Fig. 5:**
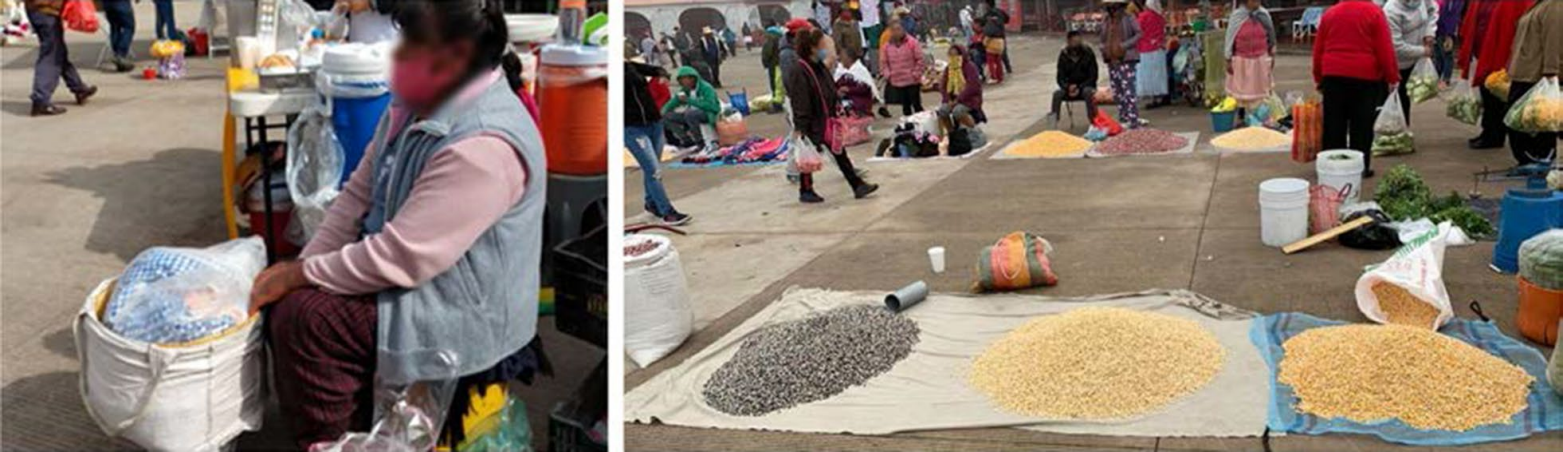
*La Placita* is a lesser *tianguis*. It is located next to the central bus station, facilitating the access and mobility of local foods. It takes place on Tuesdays and Sundays from 3:00 a.m. to 10:00 a.m. Woman selling tortillas in this *tianguis.* Photograph by Alma Lili Cárdenas Marcelo and Rafel Mier (2020)

**Fig. 6:**
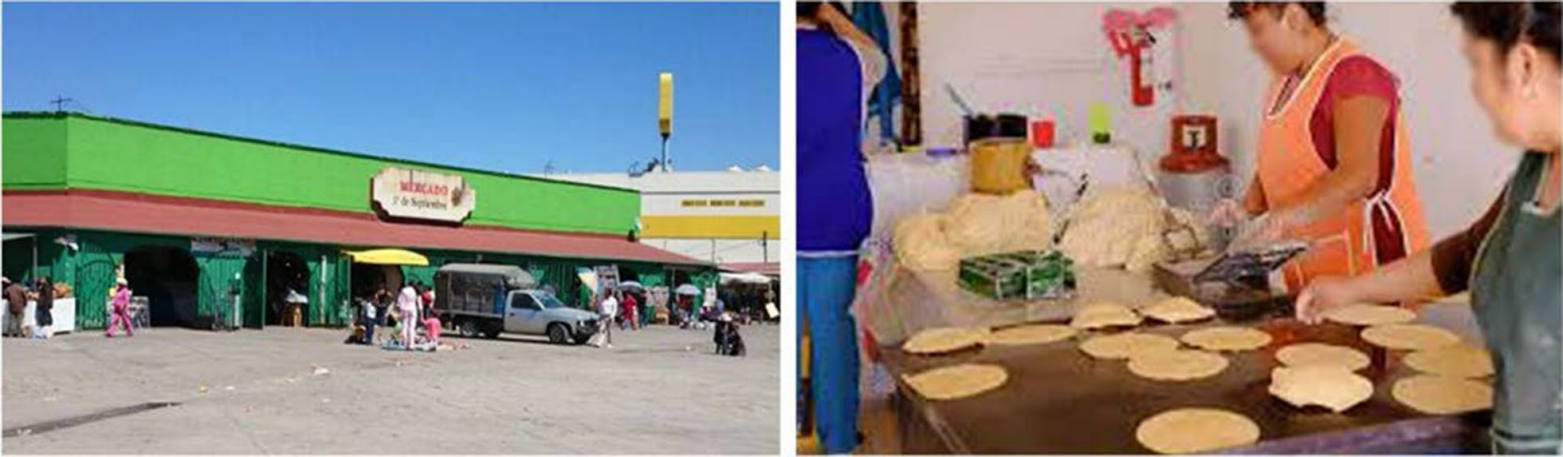
The *Mercado Municipal* within the municipality’s capital. Woman selling tortillas in this market. Photographs open access from: https://www.google.com/search?q=MERCADO+MUNICIPAL+IXTLAHUACA+DE+RAY%C3%93N&rlz=1C5CHFA_enMX896MX898&sxsrf=ALeKk02QpmoDi6lIru9ByrwHt0uPayBSOA:1614916619102&source=lnms&tbm=isch&sa=X&ved=2ahUKEwiOjLGboZjvAhUKSK0KHTVPAtkQ_AUoAnoECAQQBA&cshid=1614916649522439&biw=1234&bih=718#imgrc=9eXbC0Qe_BWX5M

At the different marketplaces, local products, but also regional and imported products are found, many of them of Asian origin [22]. In the urban areas of Ixtlahuaca, four marketplaces were identified where handmade maize tortillas are prepared and sold: (1) The Travelling *Tianguis* that takes place on Mondays, where the making and selling of tortillas is performed in open spaces in the central streets of the municipal capital (Fig. 4); (2) *La Placita,* (The Little Plaza) where tortillas made at home are sold from straw baskets covered with a cloth napkin to keep them warm, as was done in Precolumbian times [14] (Fig. 5); (3) Municipality Market, a public building with fixed daily sale schedules, administered by the local government (Fig. 6).

At present, the first two marketplaces are *tianguis* that maintain the configuration of the Prehispanic markets. A great diversity of ethnic products associated with the gastronomy of the Mazahua culture can be found there, such as *quelites* (Pigweed), mushrooms, *pulque* (fermented agave drink), handicrafts, and tortillas of various colors and sizes. Products continue to be exchanged through barter (*trueque* in Spanish) [35], thus replicating ancestral trade customs. For this reason, *tianguis* have been considered as reservoirs of natural and human biodiversity [36].

## Results and discussion

In the Mazahua culture, the remnant of the umbilical cord of a newborn girl is thrown into the stove as a symbol of her responsibility of maintaining the family unity, both through reproduction and through the livelihood related to the preparation of tortillas and other traditional foods [37]. Following the cultural tradition of assigning gender roles, three factors were found over the totality of the 36 interviews conducted. The first one is the woman-to-woman transmission of knowledge about the preparation of tortillas in the same family. The second factor is the cultural legitimacy of expanding their knowledge into public spaces. Being an exclusively female assignment, it did not result in family conflicts when some of them, in addition to making tortillas for self-consumption and in community *Fiestas*, also went to the marketplaces to sell them thus contributing to the family income. Finally, the third factor is their access to native maize varieties. Their experience has helped women develop an extensive knowledge of the use and handling of different varieties. This knowledge is vital for the understanding and conservation of the native maize diversity [20].

In general, trade carried out by women in local markets is restricted to the surroundings of the community of origin, in order not to “neglect” household responsibilities [5]. These same conditions of ethnicity and class (difficulties in accessing the means of subsistence) are observed in our study, where, addition, the analysis of gender inequalities is intertwined (childless or with children, age and sex of descendants, care of economic dependents). The most notable of these is the double workload that happens when women acquire new social responsibilities by taking over the head of the household without neglecting household chores and parenting duties. Table 1 shows that 13 of the 36 women interviewed are heads of their household. To accomplish all their tasks, these women have relied on mutual support, mainly from fellow vendors, mothers, daughters, and sisters. This sisterhood strategy guarantees, in a way, their participation and permanence in these markets. In comparison, those who contribute to income but are not head of their household have fewer such support networks on which to rely, as the husbands may not consider tortilla-selling as an essential or helpful household support activity [21].

It is nonetheless common for indigenous merchant women to trade and barter wild harvest produce such as mushrooms, edible and medicinal plants, agricultural leftovers, and maize-based products, along with selling tortillas. Moreover, tortillas not sold by the end of the day may serve as barter to secure other products they need. It is worth noting that male merchants can usually sell at more distant places, and also products not necessarily derived from maize diversity [20].

Women at *La Placita* often sell tortillas sitting on the sidewalk, without minimum safety conditions. They usually sit in small groups of three to four women as an implicit socialization strategy in these small spaces. Many of them are in the “empty nest” stage of the family life cycle. In contrast, women who sell at the Travelling *Tianguis* and the Municipality Market are usually in the “consolidation” stage of the family life cycle. The differences between these women are manifested in female support networks that help out with the care of children under the age of 11. As those who sell at the Travelling *Tianguis* lack these support networks, they have opted to sell only one day per week to reconcile their commercial ventures with their household and parenting activities, even if this means a reduction in income.

Age marks a specific cultural and social context that defines the insertion into a specific market type (Table 1). In the case of the sellers at *La Placita*, the average age is 57 years. These women grew up in a time when, although all children had access to secular, compulsory, and free primary education (established in 1867), families favored giving boys better educational opportunities, as they were considered to be the future earners [37]. This explains why 11 of the 16 interviewed women did not complete their primary education. A low education level is a factor that limits their opportunities for finding better spaces at the marketplaces to sell their products. Hence, women over 55 years of age not having finished basic education were found to sell on the sidewalk.

In the case of the Traveling *Tianguis*, women were 41 years old on average, and all of them had completed elementary school. In turn, at the Municipality Market women were 38 years old on average, with higher levels of education.

The access to education for a greater number of women generates positive and significant relationships. Still, women are not yet concretely engaged in local political participation where they can influence decision making, at least in the areas where they work.

Inequalities relative to class among women is the access to land to grow their own maize. More than half of the interviewed women (located mainly at *La Placita* and at the Travelling *Tianguis*) indicated that they do not own agricultural land, but that their husbands do and can decide what type of maize to plant (see Table 2, Types of tortillas). This option has allowed many women to prepare tortillas at a perceived lower price for a greater profit margin.

Although they consider it an “advantage” over the women that must buy maize, it is a misleading advantage, as they do not consider the costs of growing maize on their property. Women who do not have direct or indirect access to land or to maize production must buy maize in regional markets. Ordinarily, they look for hybrids produced in intensive systems, as they are cheaper than native maize varieties [21].

Paradoxically, until the restrictions on activities due to the pandemic were set in place, the women who earned the highest income were those who sold at a permanent store and bought this hybrid maize. On average, they made the biggest sales volume, and hired at least one employee. They also had the capacity to pay the monthly rent of the store, the land use fees collected by the Municipality Market administration, and other expenses such as gas, water, and electricity. In contrast, women who produce tortillas at home and sell them at *La Placita* generally use biomass fuels (wood and garbage), which represents the lowest rung on the energy ladder. Admittedly, fuel inequality and the use of inefficient technologies is an understudied indicator of energy poverty [29].

Table 2 summarizes the different tortilla sales strategies by marketplace, highlighting some inequalities related to the acquisition of corn flour, as well as the preparation and sale of handmade tortillas.

### During first period COVID-19 pandemic

Throughout 2020, the global COVID-19 pandemic has forced countries to put in place extraordinary health, social, and economic measures to reduce the massive spread and lethality of SARS-CoV2 [32]. Among these measures is the classification of activities by importance to the wellbeing of societies, giving functionality to some occupations, reorganizing others, and canceling those perceived as less necessary during confinement. Mexico established these emergent actions on March 23rd, as the country entered the most challenging quarantine period. One of the government-dictated measures was that the food sector would remain classified as an essential activity; accordingly, the food systems would continue to operate in their entirety [38], including all permanent marketplaces. On the other hand, the opening of *tianguis* was left to the interpretation of local (municipal) governments. These measures changed over the course of the pandemic and adjusted to the social, economic, and political demands of society to cope with the different waves of COVID-19 infection rates in Mexico.

The second phase of this study was conducted precisely after the first measures were implemented. They kept the population in restrictive confinement (June to August 2020). The municipal government of Ixtlahuaca did not allow the Travelling *Tianguis* to continue operating during the first 3 months of the quarantine period in Mexico (March to May 2020). By contrast the *La Placita* and Municipal Markets did not stop their commercial activities during the entire period.

This situation did not translate into greater advantages for the women who continued to sell their tortillas in *La Placita*. Before the pandemic, we observe that they experience very significant inequalities marked by old social structures. Their lives are lived in subordination because of their gender, class, ethnicity, age, and family life circle. Recent studies have shown that the predominance of norms, values and cultural beliefs regulating the social structures in traditional communities, are factors that undergo few or no transformation in the relations and roles of gender when facing economic crises or instability [31, 39].

Under such rigid structure, the conditions of vulnerability in which many Mazahua women live (low levels of education, limited access to economic resources, empty nest, energy poverty) [5, 37], become also determinant factors in reducing the possibilities to react with coping strategies when faced with the scenario of uncertainty that the pandemic brought about in its initial stage of confinement. Nevertheless, depending on the type of market where they sell their tortillas and the food needs of their households, it was viable to identify who had more room for maneuver to continue their work. For example, the women selling at *La Placita* did not develop alternative strategies to the production and sale of tortillas that would provide them with other means of sustenance. In consequence, they risked continuing to sell every day at the marketplace and on the sidewalks, despite the risk of SARS-CoV-2 infection that this represented.

During this timeframe (March–July) the poorest and oldest women experienced a marginal increase in tortilla sales, since the restrictions that conditioned them before the pandemic (production capacity and mobility, related to the weight of their baskets) continued to persist and briefly acted in their favor.

Those who do not produce their own maize were forced to increase the price of handmade tortillas by 30% (Table 2). This was because the price of hybrid maize has also increased, despite policies that aim to guarantee the supply of this basic grain at affordable prices during this time of health crisis. The price of gas and electricity has also risen, compromising the livelihood of these women at Municipality Market, who sacrifice profit margins to remain competitive, especially in the light of the fact that the price of industrialized corn flour tortillas has not increased.

On the other hand, who have school-age children have experienced other limitations that have subjected them to further class and family life cycle (reproductive period). For example, they encountered drastically reduced times at their disposal for the preparation and selling of tortillas. Some even had to abandon this activity altogether, particularly if the burden of household activities (chores and care of children or family members suffering from COVID-19) had increased. Indeed, online education and at-home learning, means they now must devote more time to their children’s learning, which restricts the ability to leave home to earn a living. As a result, many have impoverished themselves and gone into debt to have access to computers, cell phones, internet, and electricity. These debts increase with the purchase of medicines and expenses for private medical consultations to treat infected family members or themselves.

It would have been expected that women established in the Municipal Market would have faced this restrictive period with better performance due to their economic capabilities. However, they have been unable to either sustain the cost of renting and nor securing jobs. The municipal administration has not stopped collecting fees. This has subjected sellers to a new economic crisis, with debts accumulating. Some have been threatened by the marketplace management with not renewing selling permits, or even canceling valid ones.

Among the limitations of this study, besides the challenge of doing field work amid mobility restrictions, is that it was not possible to investigate an emerging market that took on importance. The sale from door of door is in fact the least explored market that appears to be a more recurrent strategy in the region of Central Mexico in the face of the health crisis.

Although this market was not analyzed due to its own mobility dynamics from door to door, two women who previously had their stalls in the Traveling *Tianguis*, they resorted to selling their tortillas in this mode to be able to obtain an income and cover the new expenses that increased at home (the cost of the internet or the purchase of electronics for online learning, medications, etc.).

Tortillas are essential in the Mexican food, and they are part of the eating habits of most people in the Mexican society, besides, the tortillas are used to prepare a great variety of Mexican (patrimonial) dishes [9, 10]. Many rural women who cook them, saw the opportunity to enter this market during periods of restricted mobility to acquire the handmade tortillas or industrial made tortillas in fixed establishments. Thanks to this emerging feminine strategy, it is highly possible that women are contributing to the food security of many rural households in Mexico, especially in the face of restriction and low mobility [39].

Certainly, food anthropology has been interest to analyze the markets where the rural and poor women sell the traditional tortillas: on the sidewalks of the cities’ streets and in the markets of Mexico [10], as well as from house to house [5]. In this past decade, the attentiveness has been accentuated since Mexican food was declared Intangible Heritage of Humanity by the UNESCO [9].

A few studies show that in this large-scale cultural movement, it is the chefs promoting the Mexican cuisine at the international level who have benefited from the work, experience, knowledge, and vulnerability conditions experienced by women who produce tortillas by hand in such kitchens as employees with the lowest staff salaries, as well as by the direct or indirect purchase of those tortillas at much lower prices than in the formal market [40]. Even so, women prefer to accept this reality than not to earn a living.

Government decisions to slow down the country's economy in order to contain the contagion did not completely stop the informal economy [32], where a large part of the sale of food takes place on the streets, including ethnic foods such as corn tortillas. It would be worth asking if economic rationality is really part of these emerging feminine strategies, because these women take the risks of contagion to carry and sell this food on the streets and at people’s homes.

At the beginning of the quarantine and the mobility restrictions, the intermediaries in the sale of tortillas disappeared in the markets researched. This along with the closure of the Traveling *Tianguis* meant a twofold blow for women that used to sell their tortillas there. In this context, these women had no income during the 3 months the market was closed. The lack of any other source of income, supplies, health services, the lack of ability to access financial resources, among other means to acquire corn or sow it, have reduced the possibilities of returning and recovering their spaces to sell in this market, which was rehabilitated since the month of August in 2020. Coupled with the increase in the prices of inputs, it is inferred that their return will demand long-term indebtedness and pre-existing gender inequalities in rural areas will deepen.

## Conclusions

The exacerbation of inequalities in Mexico before and during the COVID-19 Pandemic has made visible the problems of making quality health care accessible, the ability to sustain employment and to provide continuity in education. This study made it possible to analyze the structural gender inequalities that existed before the pandemic, which deepened, making it evident that power structures remain immovable to the detriment of women who make and sell handmade corn tortillas in different markets in central Mexico. However, this research also showed that through the intersectionality perspective, slight differences are observed among these women. It was revealed that having higher levels of education, consolidated women's networks, and being in a family life cycle childless, increase the possibilities of recovering from the health crisis and reactivating their economic activities.

It is hoped that this paper will be useful for decision-makers in their domestic and international agendas that seek to re-establish food systems tied to local traditions. In essence, ethnic foods contribute to food security with short food supply chains, not only before and during, but also after the pandemic. Undoubtedly, these decisions should focus on the rescue and strengthening of biodiversity, be it through its promotion as culinary heritage or as a domestic food security policy, but always considering the valuable and essential female contribution, where equal opportunities are guaranteed to women who live in rural areas and who sustain the native corn tortillas, as one of the most important ethnic foods in the Mexican diet.

## Data Availability

Transcripts of interviews are not available in this section. The material can only be accessed upon the express request of the applicant, as long as it is used to verify strict narratives that support the findings of this research.
